# Biomarkers of peanut allergy in children over time

**DOI:** 10.1111/all.16193

**Published:** 2024-06-18

**Authors:** Ru‐Xin Foong, George Du Toit, Ronald van Ree, Henry T. Bahnson, Suzana Radulovic, Jo Craven, Matthew Kwok, Zainab Jama, Serge A. Versteeg, Helen A. Brough, Kirsty Logan, Michael R. Perkin, Carsten Flohr, Gideon Lack, Alexandra F. Santos

**Affiliations:** ^1^ Department of Women and Children's Health (Pediatric Allergy), School of Life Course Sciences Faculty of Life Sciences and Medicine, King's College London London UK; ^2^ Peter Gorer Department of Immunobiology, School of Immunology and Microbial Sciences King's College London London UK; ^3^ Children's Allergy Service, Evelina London Children's Hospital, Guy's and St Thomas' Hospital London UK; ^4^ Department of Experimental Immunology Amsterdam University Medical Centers Amsterdam the Netherlands; ^5^ Immune Tolerance Network Benaroya Research Institute Seattle Washington USA; ^6^ Department of Women and Children's Health School of Life Course and Population Sciences, King's College London London UK; ^7^ Population Health Research Institute. St George's, University of London London UK; ^8^ St John's Institute of Dermatology King's College London and Guy's & St Thomas' NHS Foundation Trust London UK

**Keywords:** biomarkers, food allergy, mast cell activation test, peanut allergy, tolerance

## Abstract

**Background:**

Various biomarkers are used to define peanut allergy (PA). We aimed to observe changes in PA resolution and persistence over time comparing biomarkers in PA and peanut sensitised but tolerant (PS) children in a population‐based cohort.

**Methods:**

Participants were recruited from the EAT and EAT‐On studies, conducted across England and Wales, and were exclusively breastfeed babies recruited at 3 months old and followed up until 7–12 years old. Clinical characteristics, skin prick test (SPT), sIgE to peanut and peanut components and mast cell activation tests (MAT) were assessed at 12 months, 36 months and 7–12 years. PA status was determined at the 7–12 year time point.

**Results:**

The prevalence of PA was 2.1% at 7–12 years. Between 3 and 7–12 year, two children developed PA and one outgrew PA. PA children had larger SPT, higher peanut‐sIgE, Ara h 2‐sIgE and MAT (all *p* < .001) compared to PS children from 12 months onwards. SPT, peanut‐sIgE, Ara h 2‐sIgE and MAT between children with persistent PA, new PA, outgrown PA and PS were statistically significant from 12 months onwards (*p* < .001). Those with persistent PA had SPT, peanut‐sIgE and Ara h 2‐sIgE that increased over time and MAT which was highest at 36 months. New PA children had increased SPT and peanut‐sIgE from 36 months to 7–12 years, but MAT remained low. PS children had low biomarkers across time.

**Conclusions:**

In this cohort, few children outgrow or develop new PA between 36 months and 7–12 years. Children with persistent PA have raised SPT, peanut‐sIgE, Ara h 2‐sIgE and MAT evident from infancy that consistently increase over time.

## Graphical abstract

Peanut allergy (PA) remains relatively stable in late childhood (7–12‐years of age) with few children developing new PA or outgrowing PA. We aimed to observe changes in PA resolution and persistence over time. Children with persistent PA have raised SPT, peanut‐sIgE, Ara h 2‐sIgE and %CD63+ LAD2 cell activation evident from infancy that consistently increase over time. Abbreviations: Ig, immunoglobulin; LAD2, laboratory of allergic diseases 2; PA, peanut allergy; SPT, skin prick test.

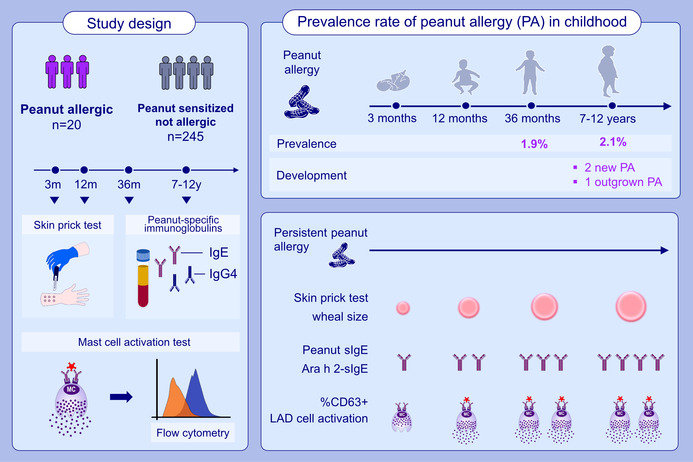

## 1 BACKGROUND

The global prevalence of IgE‐mediated peanut allergy (PA) is estimated to be between 0.2% and 4.5% depending on country,^1^ with the UK prevalence of PA in children being 2%. Approximately 20% of peanut allergic patients outgrow their allergy over time^2^, ^3^ but using biomarkers to help predict the children in whom this is more likely to occur is less understood.

The gold standard to assess the presence and the possible resolution of food allergy is the oral food challenge (OFC).^4^ However, this test comes with the risk of life‐threatening anaphylaxis, is time‐consuming, laborious, and costly, especially if multiple food challenges are needed. Other tests, such as skin prick tests (SPT), specific‐IgE (sIgE) to peanut and peanut components, are more commonly used to help establish PA diagnosis.^5^ They can also be used to understand resolution and, specifically, to help determine the right time to reintroduce an allergen back into the diet. More recently, the basophil activation test (BAT) and the mast cell activation test (MAT) have been demonstrated to have clinical utility to support the diagnosis of food allergy.^6^, ^7^ The MAT uses LAD2 mast cells (a human mast cell line) which are sensitised with patient plasma or serum, stimulated with allergen (e.g. peanut) and analysed by flow cytometry to measure CD63. Both BAT and MAT have been shown to have high specificity (ranging between 96% and 100%) in diagnosing PA^7^ but, for practical reasons, they are primarily available in the research setting.

Food allergies, such as egg and cow's milk allergies, are commonly outgrown^8^, ^9^ but for peanut and tree nut/sesame allergies, spontaneous resolution occurs less frequently. Decreasing SPT wheal size and decreasing levels of allergen‐sIgE over time are suggestive of food allergy resolution.^10^ However, the use of MAT in the context of allergy persistence or resolution has not yet been investigated.

The aims of this study were to assess the utility of different biomarkers to define trajectories of PA and PS in a general population of children over the span of a decade. We compared biomarker results between peanut allergic and peanut sensitised but tolerant children across different time points to understand their use in predicting PA persistence or resolution over time.

## 2 METHODS

### 2.1 Study population

Participants were selected from the EAT and EAT‐On studies and informed consent was obtained.^11^ The EAT study was a randomised controlled trial in which exclusively breastfeed babies were randomised to the standard introduction group (i.e. exclusively breastfeed for around 6 months and then solids were introduced as per standard UK advice) or the early introduction group (i.e. from 3 months of age infants had allergenic foods, including 4 g of peanut protein a week, introduced into their diet). The children were seen at 3‐, 12‐ and 36‐months of age with the primary outcome being IgE‐mediated food allergy between 1‐ and 3‐years of age. The EAT‐On study was conducted to establish whether the effects seen at the end of the EAT study represented a delay in food allergy onset or sustained tolerance. The EAT‐On cohort was seen between ages 7–12 years.

Each child's PA status was determined at the 7–12 years time point by either a positive OFC or a clinician‐taken history of allergic reaction to peanut and SPT ≥5 mm (if an OFC was not conducted). Tolerance was determined by a negative OFC and/or consumption of peanut regularly in the child's diet as defined by the EAT‐On study protocol (i.e. at least 3 g of peanut protein three times in the last 6 months). If the child was not consuming peanut and OFC was indeterminate or not available, a study‐specific algorithm was used to determine their allergic status (Figure S1). Peanut sensitisation was defined as having a peanut SPT ≥1 mm and/or peanut‐sIgE ≥0.1 kUA/L.^6^, ^12^, ^13^

#### 2.1.1 Skin prick tests

SPT were performed to peanut and aeroallergens on the forearm or back, using a standardised lancet (ALK‐Abello), peanut extract (ALK‐Abello), histamine 10 mg/mL or 50% glycerol, 50% buffered saline. Skin test sites were measured after 15 min as the average of the widest diameter and perpendicular of the wheal.

#### 2.1.2 Blood collection

Serum sIgE and IgG4 to peanut and peanut components (Ara h 1, Ara h 2, Ara h 3 and Ara h 8) were determined by ImmunoCAP (Thermofisher, Uppsala, Sweden). Peanut component sIgE was assayed for participants with a peanut IgE ≥0.1 kUA/L; for subjects with levels <0.1 kU/L, an imputation was performed. A ratio of Ara h 1‐sIgE, Ara h 2‐sIgE and Ara h 3‐sIgE to peanut‐sIgE was calculated to examine the distribution of each component in relation to sIgE to peanut. The median of this ratio was used to determine the imputed value for each Ara h component for patients who had peanut sIgE<0.1 kUA/L. The ratio of peanut‐specific IgG_4_:IgE was calculated after peanut‐specific IgG_4_ levels were converted from μg/L to ng/mL and peanut sIgE levels were converted from kU/L to ng/mL using the formula (IgG_4_ ÷ (IgE × 2.4)).^14^

#### 2.1.3 Peanut oral food challenge

An OFC was offered to any participant who had:

1. SPT >0 mm to peanut;
2. Previous history of PA and SPT ≥5 mm;
3. Previous history of PA whose testing suggested they might have outgrown it (i.e. SPT <5 mm or negative SPT)
4. Participants who were infrequent consumers of peanut (i.e. who consumed less than 3 g of peanut protein at least three times in the last 6 months).

All OFCs were open challenges unless there was an investigator concern about subjective symptoms, in which case a double‐blind placebo‐controlled food challenge was performed. The open challenges involved a single‐dose cumulative challenge or 6–7 dose incremental challenge if deemed to be high risk.

#### 2.1.4 Mast cell activation test

Only patients with peanut‐sIgE ≥1.0 kUA/L at 36 months had MAT performed given previously reported lower threshold of peanut‐sIgE to induce mast cell activation which can vary with intrinsic mast cell reactivity.^7^ MAT was performed as previously reported.^7^, ^13^ LAD2 cells (Laboratory of Allergic Diseases, National Institute of Allergy and Infectious Diseases) were primed with IL‐4 and incubated for 5 days before being sensitised with patient plasma or serum. The cells were stimulated with peanut extract (ALK Abello) diluted in RPMI medium (GIBCO, Paisley, UK) at two concentrations (1000 ng/mL and 10,000 ng/mL), anti‐IgE (1ug/mL, Sigma‐Aldrich, Poole, UK) and ionomycin (1 μg/mL, millipore). Cells were stained with CD63‐allophycocyanin (Biolegend, San Diego, Calif) and surface markers IgE‐PE (Biolegend), CD32‐APC, FceRI‐FITC (eBioscience, San Diego, Calif) before viability dye eFluor 450 (eBioscience) was added. Flow cytometry (CytoFLEX flow cytometer) and data was analysed using FlowJo™ v10.8 Software (BD Life Sciences, Ashland OR). MATs were performed for peanut allergic and peanut sensitised but not allergic children (PS) as defined above at the three time points (12 months, 36 months, 7–12 years), for which samples were available. Imputed MAT results were performed for participants if they had a peanut sIgE <1.0 kUA/L based on previous work that has shown MAT is dependent on sIgE levels. If sIgE is undetectable or very low, MAT will be negative which made imputation possible.

### 2.2 Statistical analysis

Data analysis was performed using Stata Statistical Software Release 17 College Stations, TX. StataCorp LLC. Mann Whitney and Kruskall–Wallis tests were performed to compare clinical characteristics and biomarkers between peanut allergic and PS children and for sub‐group analysis. Logistic regression models were also used to determine if any covariates predicted PA status at 7–12 years. Univariate analysis was performed to look at covariates affecting PA at 7–12 years followed by multivariable regression models to compare all biomarkers at each time point (i.e. peanut SPT, peanut‐sIgE, Ara h 2‐sIgE, peanut MAT at 12 months) and another to look at longitudinal comparison of single biomarkers at all three time points (i.e. peanut SPT at 12 months, 36 months, 7–12 years) in predicting PA status at 7–12 years.

## 3 RESULTS

### 3.1 Study population

A total of 947 participants were enrolled in the EAT‐On study (Figure S2) and 252 EAT participants were lost to follow up. A comparison of clinical characteristics between these two groups showed a significant difference in ethnicity with a higher percentage of those who did not return for follow up being of Asian, Black or mixed‐race descent (Table S1). There were no other significant differences between the two groups. One child was excluded as they were on peanut oral immunotherapy at the 7–12 years time point and two children with likely persistent PA had a telephone visit only, so no blood samples were collected. For the peanut  analyses, 245 children were included. There were 20 children who were peanut allergic defined by positive OFC or if no OFC was performed, they had a clinical history of reaction and peanut SPT ≥5 mm. None of the peanut allergic were consuming peanut at the 7–12 years time point. Eighteen children who were peanut allergic at 36 months were still allergic at 7–12 years (persistent PA), two were not peanut allergic at 36 months but PA at 7–12 years (new PA). There were 225 children in the PS but not allergic group of whom the tolerant status was determined by: (1) consumption of peanut regularly, (2) not consuming peanut but a negative OFC or (3) not consuming peanut, no history of reacting to peanut, no OFC but SPT between 0 and 2 mm. Only one participant in this group was peanut allergic at 36 months but no longer allergic at 7–12 years (outgrown PA). Approximately 74% (166/225) of the PS group were consuming peanut (i.e. defined as 3 g of peanut protein at least three times in the last 6 months) at 7–12 years of age.

The prevalence rate of PA in the EAT‐On cohort at 7–12 years was 2.1% (20/947) which is similar to the EAT end of study prevalence rate of 1.9% (22/1189). For the children who had clinical assessments during the EAT and EAT‐On studies, the rate of PA resolution was 5.5% (1/18). If we were to include the two children who only had a telephone visit with confirmed PA by parental report during EAT‐On and the two children who were PA during EAT but did not return for EAT‐On but assume they were still PA, the rate of resolution would be 4.5% (1/22).

The PA group were significantly more likely to have a history of eczema as well as eczema, asthma or allergic rhinitis at 7–12 years old compared to the PS group Table S2.

### 3.2 Comparing biomarkers between peanut allergic and peanut sensitised tolerant groups

PA children had significantly higher peanut SPT than PS children at 12 months, 36 months and 7–12 years (Table 1). Median peanut‐sIgE levels were also significantly different between PA versus PS groups from 3 months onwards. This was further reflected in the peanut component data with the PA group having significantly higher Ara h 1‐sIgE, Ara h 2‐sIgE, Ara h 3‐sIgE at all time points compared to the PS group as well as Ara h 6‐sIgE which was only available at the 7–12 years time point. Interestingly, at 12 months of age, the PA group already had higher Ara h 2‐sIgE levels compared to the PS group (0.3 kUA/L vs. 0.01 kUA/L, *p* < .001) and this increased and remained significantly higher over time (2.8 kUA/L vs. 0.01 kUA/L, *p* < .001 at 36 months and 16.3 kUA/L vs. 0.01 kUA/L, *p* < .001 at 7–12 years). The %CD63‐positive LAD2 cells following peanut stimulation was higher in the PA group at all time points compared to the PS group.

**TABLE 1:** Comparison of biomarkers across time between peanut allergic versus peanut sensitized not allergic patients with allergic status determined at 7–12 years. Mast cell activation following stimulation with 1000 ng/mL of peanut extract.

	Peanut allergic (*n* = 20)^a^	Peanut sensitised but not allergic (*n* = 225)^a^	*p*‐value
	Median (IQR)	Median (IQR)	
Peanut extract SPT (mm)			
3 months^b^	0 (0, 0) (*n* = 5)	0 (0, 0) (*n* = 122)	0.65
12 months	4.5 (3, 7.5) (*n* = 18)	0 (0, 0) (*n* = 223)	**<0.001**
36 months	8.5 (6.3, 10.3)	0 (0, 0) (*n* = 222)	**<0.001**
7–12 years	9.3 (7.3, 10.3)	0 (0, 0) (*n* = 223)	**<0.001**
Peanut specific IgE (kUA/L)			
3 months	0.05 (0.03, 0.2) (*n* = 18)	0.03 (0.02, 0.04) (*n* = 199)	**<0.001**
12 months	1.4 (0.64, 6.5) (*n* = 16)	0.09 (0.03, 0.3) (*n* = 197)	**<0.001**
36 months	9.3 (1.6, 46.7) (*n* = 19)	0.04 (0.02, 0.2) (*n* = 204)	**<0.001**
7–12 years	24.8 (3.6, 85.2)	0.2 (0.06, 1.0) (*n* = 216)	**<0.001**
Peanut components‐specific IgE (kUA/L) at 3 months	(*n* = 18)	(*n* = 199)	
Ara h 1	0.01 (0.01, 0.02)	0.01 (0.01, 0.01)	**<0.001**
Ara h 2	0.01 (0.01, 0.01)	0.01 (0.01, 0.01)	**<0.01**
Ara h 3	0.01 (0.01, 0.01)	0.01 (0.01, 0.01)	**<0.001**
Ara h 8	0.01 (0.01, 0.01)	0.01 (0.01, 0.01)	**<0.01**
Peanut components‐specific IgE (kUA/L) at 1 year	(*n* = 16)	(*n* = 197)	
Ara h 1	0.3 (0.04, 2.6)	0.01 (0.01, 0.01)	**<0.001**
Ara h 2	0.3 (0.06, 4.9)	0.01 (0.01, 0.01)	**<0.001**
Ara h 3	0.06 (0.02, 0.3)	0.01 (0.01, 0.03)	**<0.001**
Ara h 8	0.01 (0.01, 0.01)	0.01 (0.01, 0.01)	0.13
Peanut components‐specific IgE (kUA/L) at 3 years	(*n* = 19)	(*n* = 204)	
Ara h 1	0.1 (0.05, 1.1)	0.01 (0.01, 0.01)	**<0.001**
Ara h 2	2.8 (1.2, 24.7)	0.01 (0.01, 0.02)	<**0.001**
Ara h 3	0.6 (0.02, 3.7)	0.01 (0.01, 0.03)	**<0.001**
Ara h 8	0.03 (0.01, 0.5)	0.01 (0.01, 0.01)	**<0.001**
Peanut components‐sIgE (kUA/L) at 7‐12 years		(*n* = 216)	
Ara h 1	1.8 (0.12, 28.4)	0.01 (0.01, 0.02)	**<0.001**
Ara h 2	16.3 (1.9, 54.5)	0.01 (0.01, 0.03)	**<0.001**
Ara h 3	0.3 (0.02, 3.5)	0.01 (0.01, 0.04)	**<0.001**
Ara h 6	10.4 (1.6, 31.9)	0.01 (0.01, 0.04)	**<0.001**
Ara h 8	1.5 (0.03, 10.3)	0.01 (0.01, 0.3)	**<0.01**
Peanut‐specific IgG4 (ug/L)			
3 months	11.3 (0, 40.1) (*n* = 17)	0.02 (0, 15.1) (*n* = 188)	0.09
12 months	202.8 (74.6, 433.6) (*n* = 16)	72.0 (11.5, 421.2) (*n* = 194)	0.21
36 months	407.4 (175.7,1526.3) (*n* = 18)	190.6 (39.0, 1003.3) (*n* = 201)	0.50
7–12 years	736.2 (210.9,1482.4)	202.2 (86.6, 775.2) (*n* = 216)	**0.01**
Peanut specific IgG4/specific IgE ratios			
3 months	9.2 (0, 211.1) (*n* = 17)	0.8 (0, 187.1) (*n* = 187)	0.67
12 months	35.9 (7.1, 166.8) (*n* = 16)	276.9 (34.7, 1916.6) (*n* = 194)	**<0**.**01**
36 months	20.9 (7.5, 51.0) (*n* = 19)	1214.9 (117.5, 6948.0) (*n* = 204)	**<0**.**001**
7–12 years	11.3 (5.1, 35.1)	490.6 (71.1, 2508.7) (*n* = 216)	**<0**.**001**
MAT to peanut (%CD63+ LAD2 cells)			
3 months	0.01 (0.01, 0.01) (*n* = 17)	0.01 (0.01, 0.01) (*n* = 194)	**<0.001**
12 months	0.7 (0.01, 25.2) (*n* = 15)	0.01 (0.01, 0.01) (*n* = 195)	**<0.001**
36 months	11.5 (1.3, 30.2)	0.01 (0.01, 0.01) (*n* = 202)	**<0.001**
7–12 years	12.2 (5.0, 35.9)	0.01 (0.01, 0.01) (*n* = 178)	**<0.001**

^a^ The total *n* is denoted in () next to each individual biomarker value if it differs from the total *n* of the whole group due to missing data.

^b^ At the 3 month time point, only children in the early introduction group had SPT performed.

Bold are those that are statistically significant.

### 3.3 Comparing biomarkers between sub‐groups of peanut allergic status at the 7–12 years time point

Further sub‐group analyses were performed to assess changes in PA over time (Table 2).

**TABLE 2:** Biomarkers in participants grouped according to peanut allergic status at 7–12 years of age. Median and interquartile range are indicated. Mast cell activation following stimulation with 1000 ng/mL of peanut extract.

Peanut allergic status at the 7–12 year time point					
	Persistent peanut allergy (*n* = 18)^a^	New peanut allergy (*n* = 2)	Outgrown peanut allergy (*n* = 1)	No peanut allergy (*n* = 224)^a^	*p*‐value
Peanut SPT (mm)					
3 months^b^	0 (0, 0)	0 (0, 0)	–	0 (0, 0) (*n* = 122)	0.99
12 months	5.8 (3.5, 8) (*n* = 16)	1 (0, 2)	5.5	0 (0, 0) (*n* = 222)	**<0.001**
36 months	8.8 (7, 10.5)	1.3 (0, 2.5)	8.0	0 (0, 0) (*n* = 221)	**<0.001**
7–12 years	9.3 (7.5, 10.5)	7 (4, 10)	0	0 (0, 0) (*n* = 222)	**<0.001**
Peanut specific IgE (kUA/L)					
3 months	0.05 (0.04, 0.3) (*n* = 16)	0.03 (0.03, 0.03)	0.08	0.03 (0.02, 0.04) (*n* = 198)	**0.001**
12 months	1.5 (0.9, 7.2) (*n* = 14)	0.4 (0.1, 0.8)	1.3	0.09 (0.03, 0.3) (*n* = 196)	**<0.001**
36 months	9.5 (6.1, 46.7) (*n* = 17)	0.7 (0.2, 1.3)	0.01	0.04 (0.02, 0.2) (*n* = 203)	**<0.001**
7–12 years	30.0 (4.2, 85.4)	11.7 (2.9, 20.5)	0.02	0.2 (0.06, 0.1) (*n* = 215)	**<0.001**
Peanut components specific IgE (kUA/L)					
3 months	*n* = 16			*n* = 198	
Ara h 1	0.01 (0.01, 0.06)	0.01 (0.01, 0.01)	0.01	0.01 (0.01, 0.01)	0.39
Ara h 2	0.01 (0.01, 0.01)	0.01 (0.01, 0.01)	0.01	0.01 (0.01, 0.01)	0.78
Ara h 3	0.01 (0.01, 0.01)	0.01 (0.01, 0.01)	0.01	0.01 (0.01, 0.01)	0.32
Ara h 8	0.01 (0.01, 0.01)	0.01 (0.01, 0.01)	0.01	0.01 (0.01, 0.01)	0.98
1 year	*n* = 14			*n* = 196	
Ara h 1	0.3 (0.05, 4.0)	0.3 (0.01, 0.6)	0.06	0.01 (0.01, 0.01)	**<0.001**
Ara h 2	0.6 (0.07, 6.7)	0.1 (0.01, 0.1)	1.7	0.01 (0.01, 0.01)	**<0.001**
Ara h 3	0.1 (0.02, 0.3)	0.02 (0.01, 0.03)	0.01	0.01 (0.01, 0.03)	**<0.01**
Ara h 8	0.01 (0.01, 0.01)	0.01 (0.01, 0.01)	0.01	0.01 (0.01, 0.01)	0.85
3 years	*n* = 17			*n* = 203	
Ara h 1	0.2 (0.07, 1.1)	0.04 (0.02, 0.1)	0.01	0.01 (0.01, 0.01)	**<0.001**
Ara h 2	6.5 (1.3, 24.7)	0.03 (0.01, 0.04)	0.01	0.01 (0.01, 0.02)	**<0.001**
Ara h 3	0.9 (0.02, 3.7)	0.03 (0.03, 0.03)	0.01	0.01 (0.01, 0.03)	**<0.001**
Ara h 8	0.03 (0.01, 0.5)	0.05 (0.01, 0.09)	0.01	0.01 (0.01, 0.01)	**0.02**
7–12 years				*n* = 215	
Ara h 1	2.0 (0.1, 35.7)	1.3 (0.3, 2.4)	0.01	0.01 (0.01, 0.01)	**<0.001**
Ara h 2	19.8 (1.9, 60.5)	6.4 (0.1–12.8)	0.01	0.01 (0.01, 0.03)	**<0.001**
Ara h 3	0.3 (0.01, 3.9)	0.19 (0.2, 0.3)	0.01	0.01 (0.01, 0.04)	**<0.001**
Ara h 6	13.0 (2.3, 32.4)	1.5 (0.05, 2.9)	0.01	0.01 (0.01, 0.04)	**<0.001**
Ara h 8	0.5 (0.01, 7.9)	48.7 (2.6, 94.9)	0.01	0.01 (0.01, 0.3)	**0.011**
Peanut specific IgG4 (ug/L)					
3 months	6.1 (0, 52.8) (*n* = 15)	15.2 (15.2,15.3)	–	0.04 (0, 15.5) (*n* = 187)	0.29
12 months	202.9 (102, 436.8) (*n* = 14)	233.7 (37.1, 430.3)	–	72.1 (12.0, 421.2) (*n* = 193)	0.27
36 months	494.2 (215.9, 1800.6) (*n* = 16)	121.1 (23.3, 218.8)	99.3	196.5 (38.7, 1005.1) (*n* = 200)	0.26
7–12 years	736.2 (217.2, 1361.3)	840.9 (840.9, 1613.4)	44.3	202.5 (88.1, 776.9) (*n* = 215)	**0.04**
Peanut specific IgG4/specific IgE ratios					
3 months	4.4 (0, 63.6) (*n* = 15)	211.7 (211.1, 212.4)	–	1.3 (0, 187.1) (*n* = 186)	0.48
12 months	30.4 (6.7, 78.2) (*n* = 14)	213.2 (193.5, 232.9)	–	278.9 (36.1, 1916.6) (*n* = 193)	**<0.01**
36 months	14.7 (7.5, 21.1) (*n* = 10)	60.6 (51, 70.1)	4135.9	1210.2 (172.9, 7133.4) (*n* = 203)	**<0.001**
7–12 years	10.4 (4.5, 37.4)	21.3 (9.8, 32.8)	1044.8	484.9 (70.6, 2547.5) (*n* = 215)	**<0.001**
MAT to peanut (%CD63+ LAD2 cells)					
3 months	0.01 (0.01, 0.01) (*n* = 15)	0.01 (0.01, 0.01)	0.01	0.01 (0.01, 0.01) (*n* = 193)	0.88
12 months	4.6 (0.2, 25.2) (*n* = 13)	0.1 (0.01, 0.2)	–	0.01 (0.01, 0.01) (*n* = 195)	**<0.001**
36 months	17.8 (3.1, 30.3)	0.3 (0.3, 0.4)	0.01	0.01 (0.01, 0.01) (*n* = 201)	**<0.001**
7–12 years	12.7 (7.6, 38.9)	0.2 (0.1, 0.4)	0.5	0.01 (0.01, 0.01) (*n* = 177)	**<0.001**

^a^ The total *n* is denoted in () next to each individual biomarker value if it differs from the total *n* of the whole group due to missing data.

^b^ At the 3‐month time point, only children in the early introduction group had SPT performed.

Bold are those that are statistically significant.

**FIGURE 1:**
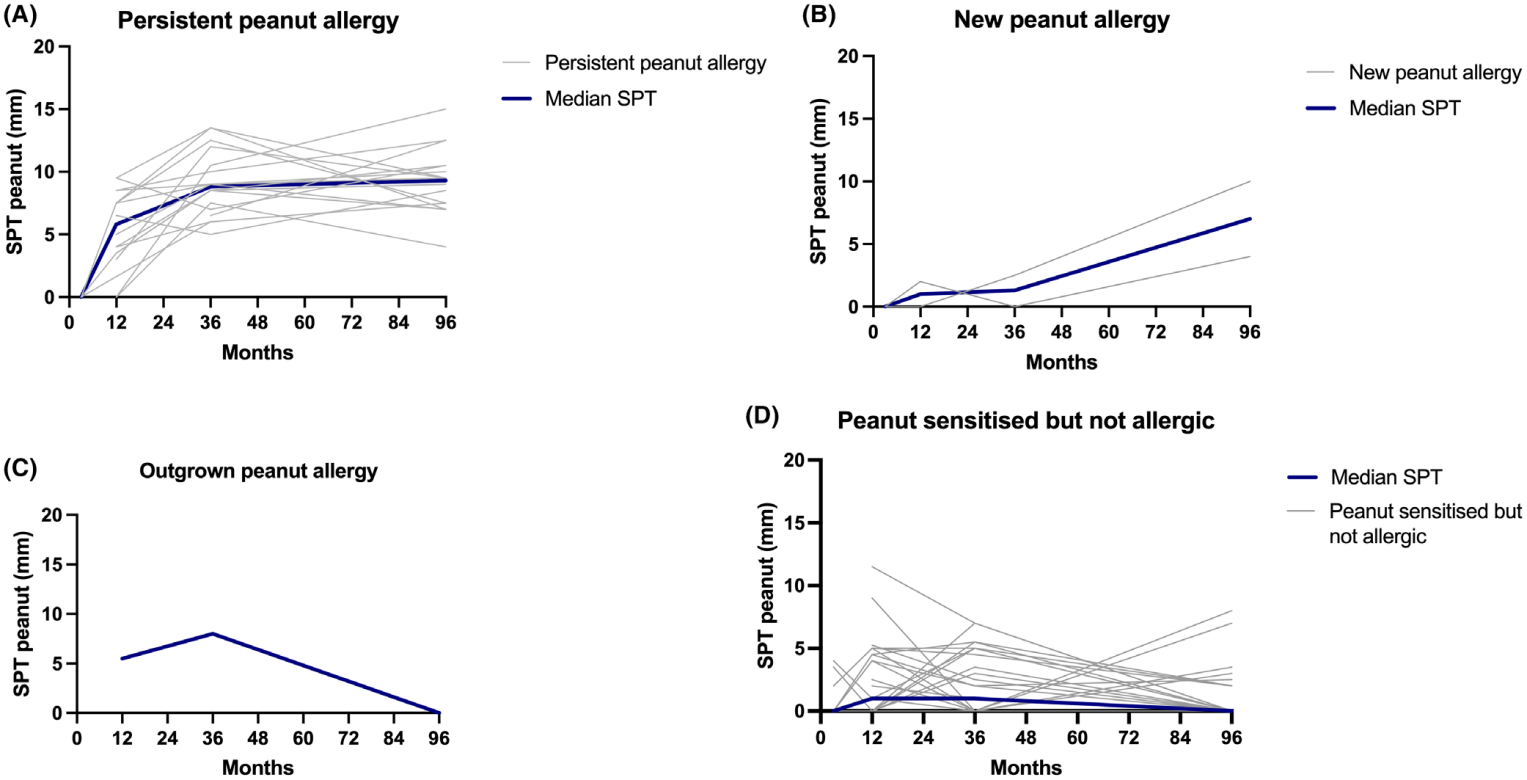
Changes in peanut SPT across time in (A) persistent PA, (B) new PA, (C) outgrown PA, (D) Peanut sensitised but never allergic (NA). The grey lines represent individual patients and dark blue line represents the median SPT at each of the time points.

**FIGURE 2:**
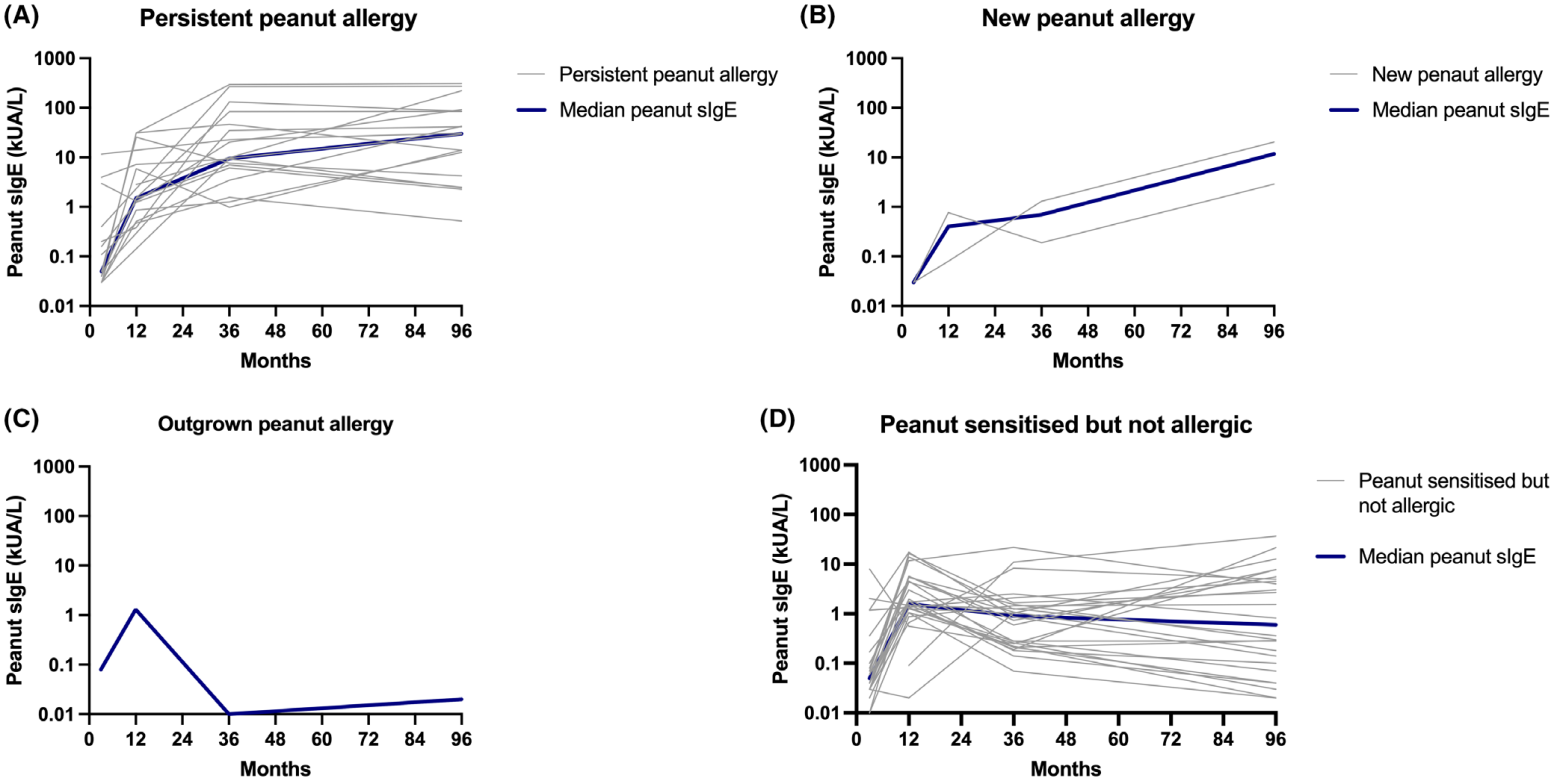
Changes in individual peanut‐specific IgE across time in (A) persistent peanut allergy, (B) new peanut allergy, (C) outgrown peanut allergy, (D) peanut sensitised but never allergic (NA). The grey lines represent individual patients, and the dark blue line represents the median specific IgE at each of the time points. IgE levels are represented on a log 10 scale.

**FIGURE 3:**
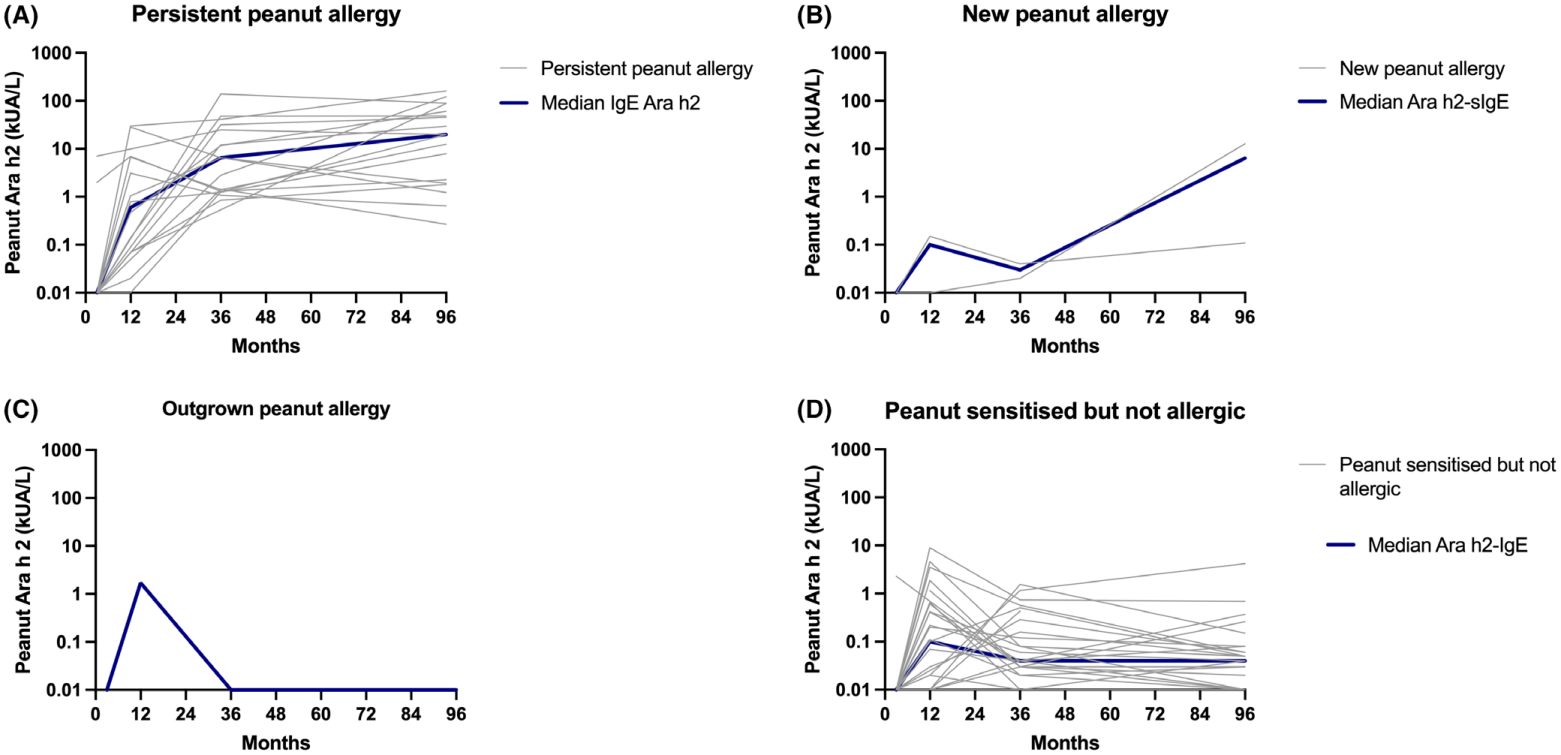
Changes in individual Ara h 2‐specific IgE across time in (A) persistent peanut allergy, (B) new peanut allergy, (C) outgrown peanut allergy, (D) peanut sensitised but never allergic (NA). The grey lines represent individual patients and he dark blue line represents the median sIgE to Ara h 2 at each of the time points. sIgE levels are represented on a log 10 scale.

**FIGURE 4:**
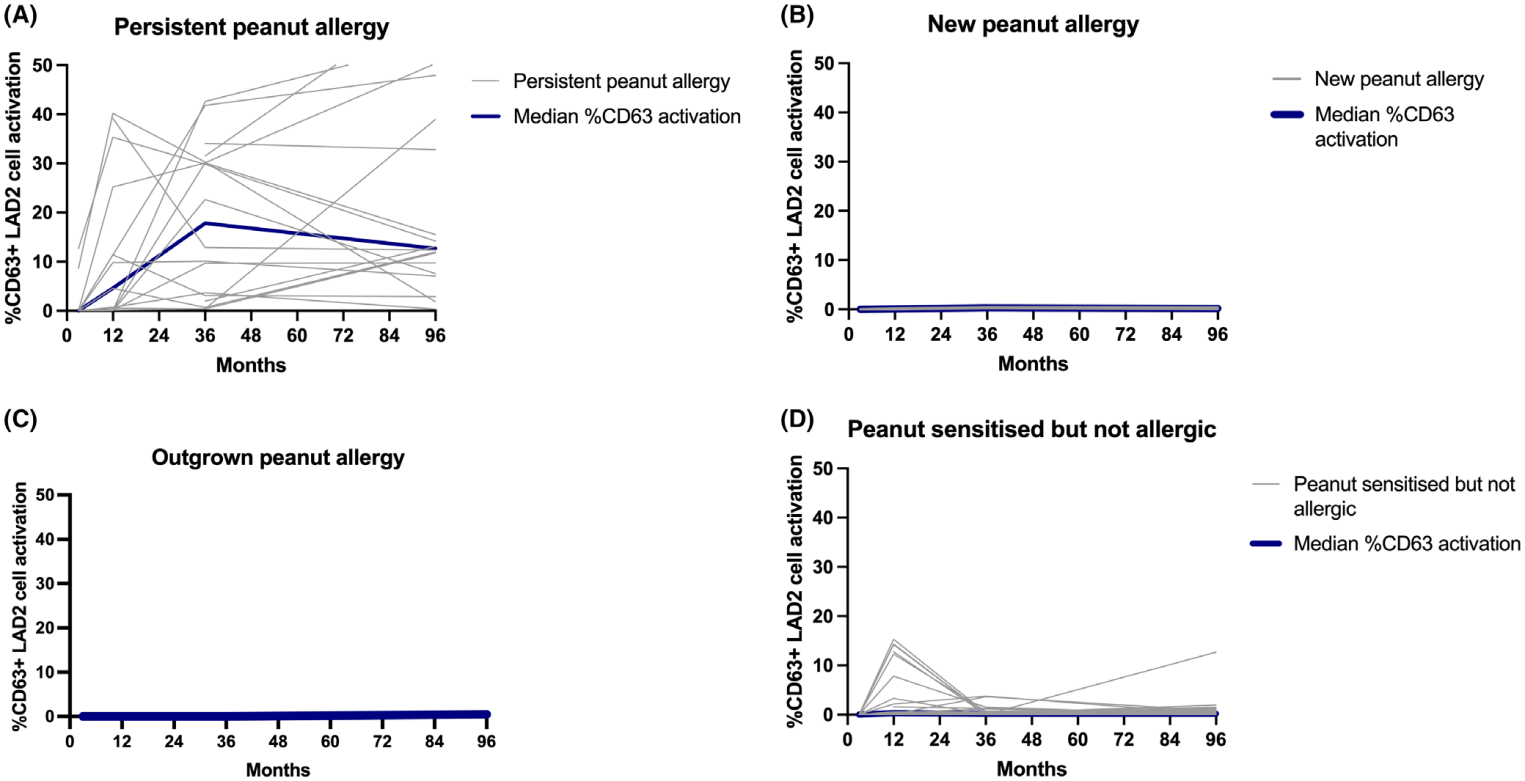
Changes in individual mast cell activation across time in (A) persistent peanut allergy, (B) new peanut allergy, (C) outgrown peanut allergy, (D) peanut sensitized but never allergic. The grey lines represent individual patients and the dark blue line represents the median % CD63+ LAD2 cells at each of the time points.

There were statistically significant differences in SPT, peanut‐sIgE, Ara h2‐sIgE and CD63+ LAD2 cells activation between the 4 groups at 12 months, 36 months and 7–12 years of age (*p* < .001) ‐ Figures 1, 2, 3, 4. Over time, children with persistent PA had SPT and peanut‐sIgE and Ara h 2‐sIgE levels that were suggestive of PA as early as 12 months of age that increased and remained persistently high; however, MAT was highest at the 36 month time point. New PA children showed increasing SPT, peanut‐sIgE and Ara h 2‐sIgE over time but the levels increased more slowly over time compared to those with persistent PA. The MAT in this group also remained low at all time points. The new PA children had an increase of Ara h 8‐sIgE levels from 36 months to 7–12 years which was significantly higher than the other three groups. The time at which this increase occurred suggests that these children developed their PA at some point between 36 months and 7–12 years of age. Both children had peanut introduced early into their diet with reports of both consuming peanut at 12 months. However, at 36 months one was no longer consuming peanut but the other was and, at 7–12 years, both were no longer consuming peanut and their biomarkers were consistent with PA. The child who outgrew PA had raised peanut SPT and peanut‐sIgE levels at 12 months of age which was consistent with PA diagnosis. However, by 36 months, although SPT was still high, their peanut‐sIgE levels had already started to decrease and by 7–12 years both SPT and peanut‐sIgE were low. In this child, MAT remained low at all time points. Children who were NA had consistently low biomarkers across time in keeping with what would be expected of non‐allergic children. There were significant differences in SPT and peanut‐sIgE between the groups at 12 months, 36 months and 7–12 years of age. Specifically, Ara h 2‐sIgE was already significantly higher (*p* < .001) in the persistent PA group at 12 months (0.6 kUA/L) which continued to increase over time (6.5 kUA/L at 36 months and 19.8 kUA/L at 7–12 years of age). This differs to the new PA group who at 36 months had undetectable Ara h 2‐sIgE (0.03 kUA/0.0 L) that then increased by 7–12 years (6.4 kUA/L) which is when they were diagnosed with PA. MAT was significantly different between the groups with higher mast cell activation occurring in persistent PA group from 12 months onwards.

### 3.4 Comparing IgG_4_ :IgE ratios between PA and PS groups

Comparison of peanut IgG4 and peanut‐specific IgG_4_:IgE ratios between PA and PS children revealed significantly higher peanut‐specific IgG_4_:IgE ratios in the PS group compared to the PA group at 12 months (276.9 vs. 35.9, *p* < .01), 36 months of age (1214.9 vs. 20.9, *p* < .001) and at the 7–12 years time point (490.6 vs. 11.3, *p* < .001) (Table 2). This was also reflected in the subgroup analysis, with the children who did not have PA because they outgrew it or never had it, as they had significantly higher peanut‐specific IgG_4_:IgE ratios compared to those who had persistent or new PA at 12 months (*p* < .01), 36 months (*p* < .001) and 7–12 years (*p* < .001) time points.

### 3.5 Biomarkers associated with peanut allergy at 7–12‐years

Logistic regression analyses were used to determine if any covariates were found to be related to having PA at 7–12 years. Demographic and clinical characteristics such as age, sex, ethnicity or history of eczema were not significantly associated with PA at 7–12 years. Univariate analyses were performed and the following covariates were found to be significant: history of eczema, eczema at 7–12 years, asthma at 7–12 years, SPT to peanut at 12 months, 36 months and 7–12 years, peanut‐sIgE at 3 years and 7–12 years, Ara h 2‐sIgE at 3 years and MAT to peanut at 1, 3 and 7–12 years (Table S3).

The biomarkers were put into multivariate regression models by time point. No peanut biomarkers were found to be significantly associated with PA at 7–12 years of age despite some of the odds ratios being high (Table S4). This suggests that the effect size over the range of variables in the model was significant. The reason why the biomarkers were not independently significant was because they were closely correlated to one another and were likely competing against each other in the model. The variation inflation factor showed moderate to high correlation between the variables. The ROC analysis of the logistic regression models for PA at the three different time points all yielded AUC ≥0.9, which suggests that these models have excellent discriminatory power in determining PA versus non‐PA cases. The small number of children who developed new PA or resolved their PA prevented further longitudinal analyses.

## 4 DISCUSSION

A good understanding of the different trajectories of PA over time is important to safely diagnose and manage PA. The prevalence of PA at both the end of the EAT study and 7–12 years was relatively stable with 1.9% at the end of the EAT study and 2.1% at the 7–12 years time‐point. There were two new cases of PA that developed after 36 months and only one child outgrew PA by 7–12 years. Children with persistent PA at 7–12 years had significantly higher levels of peanut SPT, peanut‐sIgE, Ara h 2‐sIgE and mast cell activation to peanut with these biomarkers being diagnostic of PA by 12 months, and continuing to increase over time.

At 12 months of age, the persistent PA children already had peanut SPT, peanut sIgE and Ara h 2‐sIgE levels consistent with a PA diagnosis which only continued to increase over time. Studies have reported SPT of ≥6 mm and Ara h 2‐sIgE between 0.1 and 3 kUA/L being predictors of persistent PA.^15^, ^16^ Ara h 2‐sIgE and Ara h 6‐sIgE are the peanut components most indicative of true PA,^17^ which was consistent with our findings at the 7–12 years time‐point. The IgG_4_:IgE ratios were significantly lower in the PA group and specifically in the children with persistent PA from 12 months. Overall, MAT was suggestive of PA from 12 months of age in the children who had persistent PA at 7–12 years. There were only two persistent PA patients who had plasma available from their 3 months EAT study visit who had MAT performed (Table 2). Their median CD63+ activation was 10.7% which is suggestive of PA at such an early age. The higher MAT at these time points reflected the higher levels of peanut‐sIgE levels, which we know, from previous work, induces greater mast cell activation.^7^ These changes in biomarkers demonstrate that biomarkers that are high early in childhood and increase over time are indicative of persistent PA. The PS children had significantly lower peanut SPT, peanut sIgE, Ara h 2‐sIgE from 12 months but had higher peanut‐specific IgG_4_:IgE ratios at these time points in keeping with previous literature of being tolerant to peanut.^14^ Most of the PS group (74%) were consuming peanut at 7–12 years which is likely to have contributed to these differences in biomarkers compared to the PA group, in which no children were consuming peanut at 7–12 years.

There were only two participants who developed new PA over the course of the EAT‐On study. Their peanut SPT and peanut‐sIgE were initially low until 36 months but increased over time so that by 7–12 years they were consistent with a PA diagnosis. Both these children were consuming peanut in early childhood and had a negative OFC between 12 and 36 months but both reported clinical reactions to peanut that occurred between 36 months and 7–12 years and, therefore, continued avoiding peanut by the time they were seen at the 7–12 years time point. It would be useful to have more data about their pattern of peanut consumption following the negative peanut OFC at 36 months as it is unclear whether they stopped eating peanut for a prolonged period of time and reacted on re‐exposure or if they continued to eat peanuts regularly prior to reacting. These two children had raised Ara h 8‐sIgE levels at 7–12 years which could be related to pollen cross reactivity; however, they had Ara h 2‐sIgE levels that were raised (6.4 kUA/L) and a history of clinical reaction suggestive of PA. Interestingly, MAT remained low across time in these two children even at the 7–12 years time point, which differed from the children with persistent PA who had higher MAT from 12 months onwards. A possible explanation for this lower MAT in new PA is the quality of the IgE. Hemmings et al., showed that IgE functional characteristics modify mast cell activation with higher mast cell activation resulting from higher peanut‐sIgE levels, higher specific activity, higher diversity and higher avidity of IgE for peanut.^18^ It is possible that, for those who had new PA acquired later in childhood, the allergic immune response was not fully developed and sIgE had lower levels, specific activity, diversity and avidity for peanut allergens.

There was only one child who outgrew their PA by 7–12 years, confirmed by negative OFC. Although we cannot infer conclusions on trends in biomarkers overtime based on their results alone, the patterns observed were still interesting. This child had peanut SPT and Ara h 2‐sIgE suggestive of PA at 12 months of age. Although peanut SPT remained high at 36 months, Ara h 2‐sIgE was undetectable. Their peanut‐sIgE level was lower at all time points with a peak of 1.3 kUA/L at 12 months and then was undetectable by 36 months and remained so until 7–12 years. The peanut‐specific IgG_4_:IgE ratio was also high at the 36 months and 7–12 years time points which is consistent with tolerance to peanut, as seen in previous studies.^14^

Our study is unique in that it looks at the changes in PA in a population‐based cohort of children over the span of a decade. The longitudinal nature of this study and the availability of biomarkers at the different time points helps to explain how PA is largely stable in later childhood. Our data demonstrates that high biomarkers in early childhood are associated with PA persistence, which is consistent with previous findings.^16^ MAT has high specificity in identifying children who will clinically react to peanut.^19^ This is the first study looking at MAT over time. For children with persistent PA, mast cell activation was detectable by 12 months. The utility of MAT was limited in children with very low levels of peanut‐sIgE, like those who developed or resolved their PA.

The major limitation of this study is the small number of children in the sub‐group analysis. Only two children developed new PA and two child outgrew PA which makes it difficult to draw conclusions about these subgroups. We had hoped to compare biomarkers predicting resolution of PA with persistence of PA but this was not possible in this cohort. There was also missing biomarker data in the baseline 3 months SPT (i.e. these were not performed for children randomised to the standard introduction group), peanut component‐sIgE data (i.e. was only performed if peanut sIgE >0.1 kUA/L) and MAT. We were able to impute the peanut component‐sIgE and MAT data based on sIgE levels but there were still some children who did not have data available if sIgE was missing. Also, as the children were all recruited from the EAT‐On Study, definitions for allergic status and tolerance were based on the study protocol to allow for consistency in the data analysis. In an ideal setting, all children selected for the biomarker work would have had OFC to confirm their PA status at 7–12 years of age. Another limitation is that of the 252 children lost to follow up there was a significantly higher percentage of non‐Caucasian children who we know have a higher rate of food allergies compared to Caucasian children. However, this is unlikely to have significantly influenced the rate of PA or the rate of resolution in the entire population because PA has been shown to develop earlier in non‐Caucasian children compared to Caucasian children.^20^ It is also important to acknowledge that most of the children with PA were in the standard introduction group of the original EAT study. As very few children who developed PA at age 36 months had been eating peanut before 6 months, it is difficult to argue that the intervention of early peanut introduction was responsible for the low rate of resolution. This likely represents a small numbers effect.

This data is applicable to other UK settings, as the participants were recruited from a general UK population with no risk factors for food allergy (i.e. a low risk cohort). One of the main criteria was that the mothers were planning to exclusively breastfeed for at least the first 3 months of life. The study was conducted in a tertiary allergy centre and therefore there was some possibility of bias in the participants coming from parents with atopic background. If this were a concern, one would have expected to see a higher rate of peanut and other food allergies in the EAT population. However, the rate of PA observed in our cohort was 1.9% during the EAT study and 2.1% in EAT‐On which is similar to previous rates of PA reported in school children in the UK.^21^, ^22^

To conclude, the rate of PA in this cohort of children was 2.1% at 7–12 years. Children with PA had significantly higher SPT, peanut‐sIgE, Ara h 2‐sIgE and MAT compared to PS children from 12 months onwards. For those who developed new PA or outgrew their PA, the timing at which this happened likely occurred between 36 months and 7–12 years of age, but small numbers and low biomarkers prevented additional conclusions.

## AUTHOR CONTRIBUTIONS

RF, GdT, SR, JC, KL, HB were investigators on the EAT‐On study team who had roles in the recruitment, data collection and statistical analysis of the main study. RvR and SV performed the blood sIgE and IgG4 testing in their laboratory for the EAT and EAT‐On studies. MP, GL, CF, KL, SR were leading members of the original EAT study in terms of protocol development, study procedures, patient recruitment, data collection and analysis which was used in this study. RF, AFS and GL designed this specific study and AFS obtained funding for the mast cell activation test. RF performed and analysed the mast cell activation tests with assistance from MK and ZJ, under supervision of AFS. RF performed the statistical analysis and wrote the first version of the manuscript, under supervision of AFS and GL. All authors critically reviewed the manuscript and approved the final version.

## 6 FUNDING INFORMATION

The EAT and EAT‐On studies were supported by grants from the Food Standards Agency (T07051), the Medical Research Council (MC_G1001205), Action Medical Research (GN2251) and the Food Allergy Research & Education (RE14333). The mast cell activation tests were supported by the Medical Research Council (MR/T032081/1, MR/M008517/1) and the Department of Health via the National Institute for Health Research (NIHR) comprehensive Biomedical Research Centre award to Guy's & St Thomas' NHS Foundation Trust in partnership with King's College London and King's College Hospital NHS Foundation Trust. The clinical trials unit is supported in part by the National Peanut Board, Atlanta.

## CONFLICT OF INTEREST STATEMENT

**GdT** reports grants from National Institute of Allergy and Infectious Diseases (NIAID, NIH), Food Allergy & Research Education (FARE), MRC & Asthma UK Centre, UK Department of Health through NIHR, Action Medical Research and National Peanut Board. Scientific Advisory Board member Aimmune. Investigator on pharma‐sponsored allergy studies (Aimmune, and DBV Technologies). Scientific advisor to Aimmune, DBV and Novartis. **RvR** consults for HAL Allergy BV, Citeq BV, Angany Inc, Reacta Healthcare, Mission MightyMe and has equity in Angany Inc. **SR** reports grants from National Institute of Allergy and Infectious Diseases (NIAID, NIH) to cover parts of a research salary. **HAB** reports grants from National Institute of Allergy and Infectious Diseases (NIAID, NIH), and speaker fees from DBV Technologies, outside of the submitted work. **CF** is Chief Investigator of the UK National Institute for Health Research‐funded TREAT. (ISRCTN15837754) and SOFTER (Clinicaltrials.gov: NCT03270566) trials as well as the UK‐Irish Atopic eczema Systemic Therapy Register (A‐STAR; ISRCTN11210918) and a Principle Investigator in the European Union (EU) Horizon 2020‐funded BIOMAP Consortium (http://www.biomap‐imi.eu/). He also leads the EU Trans‐Foods consortium and directs the Global Atopic Dermatitis Atlas (www.atopicdermatitisatlas.org). His department has received funding from Sanofi‐Genzyme and Pfizer for skin microbiome work. He has also received compensation from the British Journal of Dermatology (reviewer and Section Editor) and EuroGuiDerm (guidelines lead). **GL** reports grants from National Institute of Allergy and Infectious Diseases (NIAID, NIH), other from Food Allergy & Research Education (FARE), other from MRC & Asthma UK Centre, other from UK Department of Health through NIHR, other from National Peanut Board (NPB), other from The Davis Foundation, during the conduct of the study; shareholder in DBV Technologies, and Mighty Mission Me, personal fees from Novartis, personal fees from Sanofi‐Genyzme, personal fees from Regeneron, personal fees from ALK‐Abello, personal fees from Lurie Children's Hospital, outside the submitted work. **AFS** reports grants from Medical Research Council (MR/M008517/1; MC/PC/18052; MR/T032081/1), Food Allergy Research and Education (FARE), the Immune Tolerance Network/National Institute of Allergy and Infectious Diseases (NIAID, NIH), Asthma UK (AUK‐BC‐2015‐01), BBSRC, Rosetrees Trust and the NIHR through the Biomedical Research Centre (BRC) award to Guy's and St Thomas' NHS Foundation Trust, during the conduct of the study; personal fees from Thermo Scientific, Nestle, Novartis, Allergy Therapeutics, Buhlmann, IgGenix, as well as research support from Buhlmann, IgGenix and Thermo Fisher Scientific through a collaboration agreement with King's College London. All the other authors have nothing to disclose.

## Supporting information


Data S1:

## Data Availability

The data that support the findings of this study are available on request from the corresponding author. The data are not publicly available due to privacy or ethical restrictions.
