# Supplementary material for: Biomarkers of peanut allergy in children over time
Source: Allergy. 2024 Jun 18;79(10):2775–86. doi: 10.1111/all.16193 (PMC11875691; doi:10.1111/all.16193)
Supplement: Supplementary file 1 — Data S1: [file ALL-79-2775-s001.docx]

**Supplementary appendix**


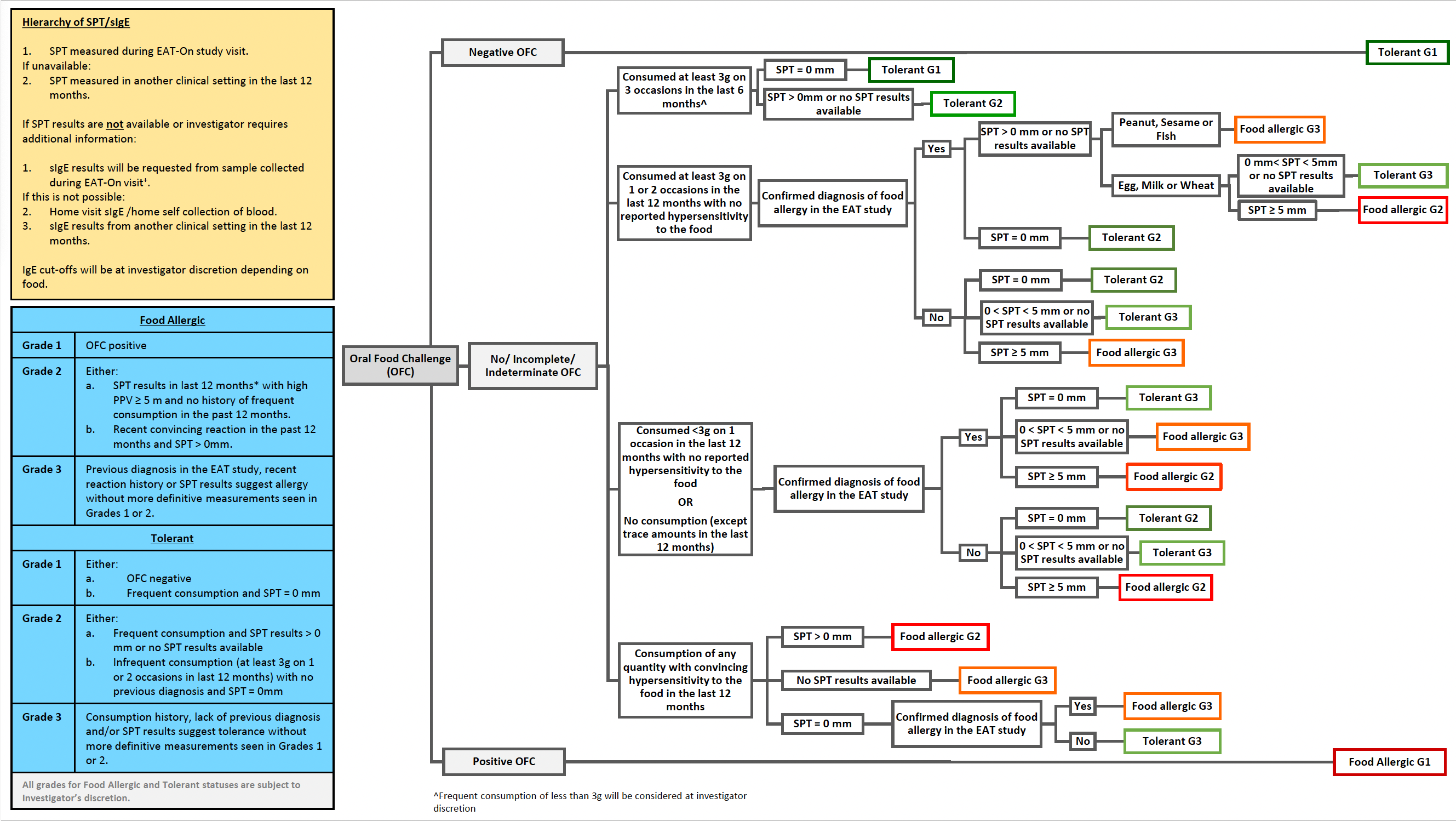


**Figure S1: Algorithm to determine food allergy versus tolerance for EAT-On study participants**


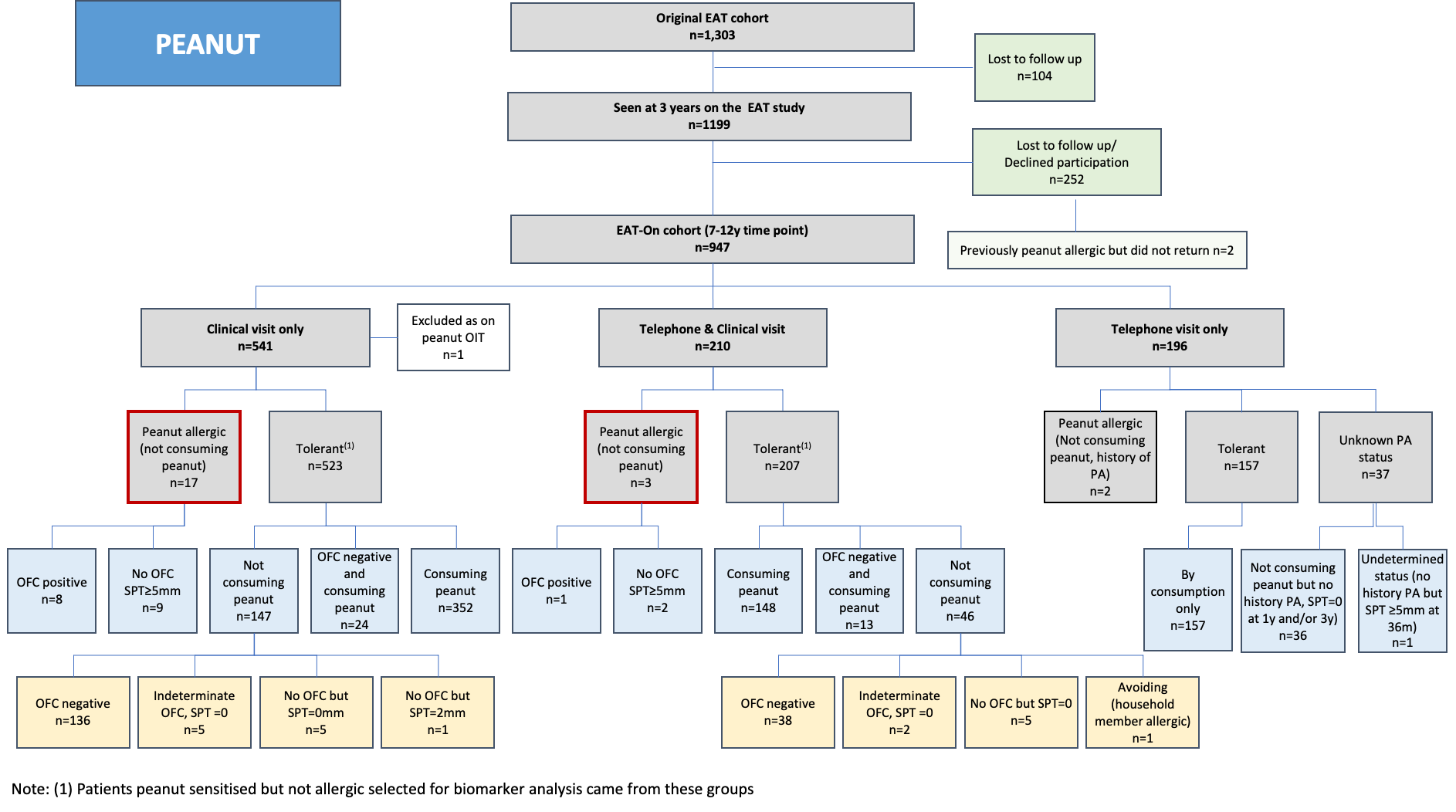


**Figure S2:** **Determining peanut allergy status for the entire EAT-On Cohort.** This flow diagram details how peanut allergic status was determined based on patient encounters during the EAT-On Study. Patients had a clinical and/or telephone visit and were found to be peanut allergic or tolerant based on oral food challenge and/or consumption (regular consumption was defined as eating 3 grams of peanut protein at least 3 times in the last 6months). If neither OFC nor consumption data were available, SPT<5mm was utilized to determine tolerance. The red box indicates peanut allergic children who were included in the biomarker analysis. [1] Represents where the patients who were peanut sensitised and not allergic were selected from for the biomarker analysis.

**Table S1: Lost to follow up - comparison of participants who were not seen on EAT-On to those seen on EAT-On**

|  | **All participants evaluated in EAT-On (n=947)** | **Participants not evaluated on EAT-On (lost to follow up) (n=252)** | **P value** |
| --- | --- | --- | --- |
| **Age mean years** (SD) | 8.8 (1.3) | **--** | -- |
| **Gender** n (%)  Female | 480 (50.7) | 111 (44.1) | p=0.06 |
| **Ethnicity** n (%)  White Caucasian  Mixed  Asian  Black  Chinese or other ethnic group | 828 (87.4)  77 (8.1)  14 (1.5)  20 (2.1)  8 (0.8) | 201 (79.8)  30 (11.9)  8 (3.2)  12 (4.8)  1 (0.4) | **p<0.01** |
| **Maternal education at EAT study time point** n (%)  16 years or younger  17-18 years old  19 years or older  Still studying | 42 (4.4)  120 (12.7)  766 (80.9)  19 (2.0) | 7.9 (20)  11.9 (30)  77.0 (194)  8 (3.2) | p=0.09 |
| **Paternal education at EAT study time point** n (%)  16 years or younger  17-18 years old  19 years or older  Still studying | 125 (13.2)  165 (17.4)  630 (66.5)  27 (2.9) | 46 (18.3)  47 (18.7)  150 (59.5)  3.6 (9) | p=0.13 |
| **Any history of atopic disease during the EAT study** n (%)   - History of atopic dermatitis/eczema - History wheeze - History of rhinitis | 52.3 (496)  44.7 (423)  17.3 (164)  33.1 (301) | 50.4 (127)  40.9 (103)  17.1 (43)  0 (0) | p=0.56  p=0.28  p=0.92  -- |
| **Eczema SCORAD severity – 3 months** n (%)  0 (Absent)  1 (Mild)  2 (Moderate) | 712 (75.2)  180 (19.0)  54 (5.7) | 193 (76.7)  46 (18.3)  13 (5.2) | p=0.88 |
| **Eczema SCORAD severity – 1 year**** n (%)  0 (Absent)  1 (Mild)  2 (Moderate) | n=912  667 (73.6)  195 (21.5)  50 (5.5) | 161 (77.8)  38 (18.4)  3.9 (8) | p=0.35 |
| **Eczema SCORAD severity – 3 years**** n (%)  0 (Absent)  1 (Mild)  2 (Moderate) | n=937  738 (78.8)  136 (14.5)  63 (6.7) | 202 (80.2)  32 (12.7)  18 (7.1) | p=0.75 |

**Table S2: Comparison of clinical characteristics between peanut allergic vs peanut sensitized not allergic children at 7-12y**

|  | Peanut allergic  (n=20)* | Peanut sensitised but not allergic (n=225)* | P value |
| --- | --- | --- | --- |
| Mean age years (SD) | 8.5 (0.5) | 8.4 (1.2) | 0.07 |
| Female sex n (%) | 8 (40) | 115 (51.1) | 0.47 |
| Ethnicity n (%)   - White Caucasian - Mixed - Asian/Asian British - Black/Black British - Chinese or other ethnic group | 14 (70)  4 (20)  1 (5)  0 (0)  1 (5) | 184 (81.8)  22 (9.8)  6 (2.7)  9 (4.0)  4 (1.8) | 0.46 |
| History of eczema n (%) | 18 (90) | 134 (59.6) | **<0.01** |
| Ecezma at 7-12y n (%) | 14 (70) | 70 (31.3) (n=224) | **<0.001** |
| Asthma at 7-12y n (%) | 9 (45) | 35 (15.7) (n=223) | **<0.01** |
| Allergic rhinitis at 7-12y n (%) | 13 (65) | 98 (44.0) (n=223) | **0.05** |
| *The total n is denoted in () next to each individual biomarker value if it differs from the total n of the whole group due to missing data. | | | |

**Table S3: Univariate analysis looking at the covariates affecting peanut allergy at 7-12 years of age**

| Independent variable | Odds ratio | Std error | p> \|z\| | 95% CI | AUC |
| --- | --- | --- | --- | --- | --- |
| Sex (female) | 0.72 | 0.33 | 0.47 | 0.3, 1.8 | 0.4587 |
| Ethnicity (Non-Caucasian) | 1.80 | 0.92 | 0.25 | 0.7, 4.9 | 0.5517 |
| History of eczema | 6.45 | 4.88 | **0.01** | 1.5, 28.4 | 0.6546 |
| Eczema at 7-12 years | 5.5 | 2.77 | **0.001** | 2.0, 14.8 | 0.7009 |
| Asthma at 7-12 years | 4.03 | 1.93 | **<0.01** | 1.6, 10.3 | 0.6358 |
| Allergic rhinitis at 7-12 years | 2.55 | 1.23 | **0.05** | 1.0, 6.6 | 0.6136 |
| SPT at 1 year | 1.69 | 0.15 | **<0.001** | 1.4, 2.0 | 0.8769 |
| SPT at 3 years | 2.30 | 0.34 | **<0.001** | 1.7, 3.1 | 0.9654 |
| SPT at 7-12 years | 2.48 | 0.39 | **<0.001** | 1.8, 3.4 | 0.9671 |
| Peanut sIgE 1 year (log10) | 6.66 | 2.45 | **<0.001** | 3.2, 13.7 | 0.8989 |
| Peanut sIgE 3 years | 20.19 | 11.67 | **<0.001** | 6.5, 62.7 | 0.9751 |
| Peanut sIgE 7-12 years | 14.85 | 7.33 | **<0.001** | 5.6, 39.1 | 0.9438 |
| Peanut rAra h 2 1 year | 5.21 | 1.63 | **<0.01** | 2.8, 9.6 | 0.8734 |
| Peanut rAra h 2 3 years | 11.95 | 5.41 | **<0.001** | 4.92, 29.0 | 0.9777 |
| Peanut rAra h 2 7-12 years | 8.42 | 3.18 | **<0.001** | 4.0, 17.6 | 0.9940 |
| MAT peanut 1 year | 1.21 | 0.06 | **<0.001** | 1.1, 1.3 | 0.8320 |
| MAT peanut 3 years | 3.67 | 1.22 | **<0.001** | 1.9, 7.0 | 0.9879 |
| MAT peanut 7-12 years | 1.79 | 0.25 | **<0.001** | 1.4, 2.4 | 0.9851 |

**Table S4:** Multivariable logistic regression analyses looking at the covariates affecting peanut allergy determined at 7-12 years of age by time point over time

| Independent variable | Odds ratio | Std error | p > \|z\| | 95% CI | VIF | AUC |
| --- | --- | --- | --- | --- | --- | --- |
| Multivariable analysis of data at 1 year time point | | | | |  |  |
| SPT at 1 year | 1.10 | 0.17 | 0.53 | 0.8, 1.5 | 3.50 | 0.9135 |
| Peanut sIgE 1 year (log10) | 1.65 | 1.10 | 0.45 | 0.4, 6.1 | 2.15 |  |
| Peanut rAra h2-sIgE 1 year (log10) | 2.63 | 1.81 | 0.16 | 0.7, 10.2 | 4.86 |  |
| MAT peanut 1 year | 1.02 | 0.06 | 0.70 | 0.9, 1.1 | 1.74 |  |
| Multivariable analysis of data at 3 year time point | | | | |  |  |
| SPT at 3 years | 1.57 | 0.33 | 0.03 | 1.0, 2.4 | 2.74 | 0.9849 |
| Peanut sIgE 3 years (log10) | 1.23 | 1.12 | 0.82 | 0.2, 7.3 | 3.31 |  |
| Peanut rAra h2-sIgE 3 years (log10) | 3.45 | 3.05 | 0.16 | 0.6, 19.5 | 5.96 |  |
| MAT peanut 3 years | 1.16 | 0.28 | 0.55 | 0.7, 1.9 | 2.41 |  |
| Multivariable analysis of data at 7-12 year time point | | | | |  |  |
| SPT at 7-12 years | 1.98 | 1.05 | 0.20 | 0.7, 5.6 | 4.63 | 0.9977 |
| Peanut sIgE 7-12 years (log10) | 0.46 | 0.55 | 0.51 | 0.05, 4.7 | 2.15 |  |
| Peanut rAra h2-sIgE 7-12 years (log10) | 3.62 | 6.15 | 0.45 | 0.1, 101.5 | 7.13 |  |
| MAT peanut 7-12 years | 1.42 | 0.38 | 0.20 | 0.8, 2.4 | 2.24 |  |
